# Cognitive and psychological factors associated with severe insomnia in Brazilian women: a cross-sectional study

**DOI:** 10.1186/s41155-022-00243-x

**Published:** 2022-12-23

**Authors:** Renatha El Rafihi-Ferreira, Marwin Machay Indio do Brasil do Carmo, Lucas Bassolli, Rosa Hasan, Isabela Mayumi Nishino Aizawa, Andrea Cecilia Toscanini

**Affiliations:** 1 Instituto de Psiquiatria, Universidade de São Paulo, 785 Doutor Ovídio Pires de Campos Street, São Paulo, SP 05403-903 Brazil; 2 Hospital das Clínicas, Faculdade de Medicina, Universidade de São Paulo, 785 Doutor Ovídio Pires de Campos Street, São Paulo, SP 05403-903 Brazil

**Keywords:** Sleep, Insomnia, Beliefs about sleep, Psychological inflexibility, Women

## Abstract

Insomnia is the most prevalent sleep disorder in women. Sociodemographic, cognitive, and psychological factors may contribute to its severity. This study evaluated factors associated with severe insomnia in women with insomnia complaints. We evaluated 530 women aged 18–59 (mean = 40.5, *SD* = 10.2), who experienced insomnia complaints, using self-report instruments. Severe insomnia was defined as a score above 21 on the Insomnia Severity Index. Age, marital status, educational level, depression and anxiety, psychological inflexibility, and beliefs about sleep were assessed as potential factors associated with severe insomnia. Simple and multivariate analyses were conducted using binary logistic regression. Low education level (odds ratio; *OR* = 1.85 [1.27–2.69]), depression (*OR* = 2.17 [1.27–3.81]), psychological inflexibility (*OR* = 1.05 [1.02–1.08]), and dysfunctional beliefs about sleep (*OR* = 1.04 [1.02–1.06]) were factors associated with severe insomnia scores in the multiple logistic regression model. These findings are important from a public health perspective, because behavioral strategies designed to treat insomnia with a focus on cognitive and psychological factors are low-cost treatments and may help improve sleep quality in women, which also influences mental health.

## Introduction

Chronic insomnia can be defined as a difficulty in falling asleep, maintaining sleep throughout the night, or waking up earlier than desired, given the opportunity and an adequate sleep environment. It must be accompanied by daytime symptoms and not be better explained by another disease or medication that can justify the condition. Moreover, the symptoms should occur thrice per week for at least 3 months (American Academy of Sleep Medicine, 2014).

Chronic insomnia can occur independently or in combination with other medical or psychiatric disorders (Morin & Benca, 2012). The literature reports adverse effects of insomnia on mental and physical health, including metabolism and productivity; the condition is also strongly associated with an increase in health spending (Lian et al., 2015). Additionally, chronic insomnia is associated with susceptibility to infections, obesity, and an increased risk of hypertension and infarction (Grandner et al., 2012). Psychological conditions such as anxiety, depression, and stress may also be present in patients with insomnia (Staner, 2010). Furthermore, these individuals have a 6.7 times greater chance of needing medical treatment for a health reason (Kryger et al., 2010). The economic burden of insomnia is substantial, considering decreased productivity and increased healthcare utilization (Buysse, 2013); therefore, the cost of untreated insomnia is substantially higher than that of its treatment (Daley et al., 2009).

The prevalence of chronic insomnia in the general population is 10% (Kronholm et al., 2015). However, epidemiological research shows that sleep dissatisfaction is even more frequent and ranges from 10 to 48% (Paparrigopoulos et al., 2010). Studies consistently report a higher prevalence of insomnia in women, with an incidence of nearly two-fold compared to men (Mallampalli & Carter, 2014; Sidani et al., 2019; Zhang & Wing, 2006). The interactions among biological (hormonal cycle and age), psychiatric/psychological (anxiety and depression), social (role in society, work and home responsibilities, socioeconomic condition/vulnerability), and cognitive (beliefs about sleep and psychological inflexibility) factors can contribute to the etiology, maintenance, and severity of insomnia in women.

Aging plays a role in biological and social contexts and is independently associated with sleep fragmentation, insomnia symptoms, circadian derangements, and sleep architectural changes such as lighter sleep, reduced sleep efficiency, increased wake after sleep onset (WASO), decreased slow wave sleep (SWS), and rapid eye movement (REM) sleep (Ohayon et al., 2004). All these factors can be associated with insomnia complaints, especially considering the perception of the importance of good sleep quality that happens over time (Ohayon et al., 2004). A study conducted in the Brazilian population showed that insomnia and light sleep were more observed in older subjects (male and female), including an association between lower educational level and lower family income with insomnia (Hirotsu et al., 2014).

Among social factors, income and education levels seem to play a role in the insomnia context. Some studies showed that the prevalence of insomnia was higher in individuals with lower incomes and lower education; however, these results were contradictory if the same factors were analyzed as independent risk factors for insomnia symptomatology, thus leading to the belief that other parallel aspects such as age may influence these results (Ohayon, 2002).

A recent cross-sectional study carried out in Spain identified certain characteristics of the local insomnia population. The sample consisted of 1563 participants aged between 18 and 80, initially screened through the Insomnia Severity Index (ISI) followed by a face-to-face clinical interview. The results revealed women, the widowed and divorced, and retired and unemployed as the population more likely to present insomnia. Anxiety and depression were also present in 37.9% and 21.1% of the participants, respectively (Torrens et al., 2019).

Insomnia is commonly comorbid with several psychiatric disorders, particularly anxiety and depression, and the literature indicates that sleep disturbances are more severe in the presence of comorbid conditions (Sarsour et al., 2010). Recent studies consider sleep disturbance as an independent diagnostic entity that may precipitate the onset of depressive disorder and conclude that improving sleep reflects positively on the improvement of depression (McCall et al., 2010). A recent review discussed the role of sleep disturbance as a prodromal symptom, which can predict the occurrence and outcome of depression. The same review highlighted the importance of treating sleep disturbance before, during, and after depression (Fang et al., 2019). Taylor et al. (2005) developed a cross-sectional and retrospective study comparing depression and anxiety in different insomnia types. Their sample comprised 772 subjects (men and women) evaluated by self-report measures of health, sleep, depression, and anxiety. The study demonstrated that insomnia participants presented greater levels of depression and anxiety than their peers not having insomnia and were 9.82 and 17.35 times as likely to have clinically significant depression and anxiety, respectively.

Although cognitive factors exert an important influence on the perpetuation of insomnia, the high prevalence of insomnia in women has been attributed to biological factors, with only a few studies focusing on cognitive factors. In this regard, there are two cognitive processes involved in insomnia etiology and maintenance: the process of interpretation and that of sleep interference (Lundh, 2005).

The interpretation process refers to the role of beliefs and cognitions about sleep as a cause of unsatisfactory sleep (Harvey, 2002). Morin et al. (2007) suggest that sleep-related cognitions such as faulty expectations, beliefs, perceptions, and excessive worry play a relevant mediating role in perpetuating and exacerbating insomnia. Some people with insomnia suffer unrealistic expectations about their sleep requirements and fear the potential consequences of insomnia on their daytime functioning. These dysfunctional beliefs about sleep increase pre-sleep arousal and contribute to the vicious cycle of insomnia (Morin & Espie, 2003). For instance, Morin et al. (1993) evaluated beliefs and attitudes about sleep in people with and without insomnia. The outcomes showed that insomniacs endorsed stronger beliefs about the negative consequences of insomnia, expressed more hopelessness about the fear of losing control of their sleep, and experienced more helplessness about its unpredictability. Therefore, these beliefs about sleep may be instrumental in perpetuating insomnia. Habits and beliefs about sleep are the therapeutic focus of cognitive therapy (Morin et al., 2007). Conversely, the sleep interference process encompasses physiological and cognitive arousal, which can arise with emotional experience activation or the effect of cognitive stress responses (Espie, 2002). This arousal can include avoidance of private experiences, inflexibility, and attempts to suppress thoughts (McCracken et al., 2011).

Recent studies have demonstrated the association between psychological inflexibility and the severity of insomnia and sleep problems (Kato, 2016; Kato, 2020; McCracken et al., 2021). For instance, the study conducted by Kato (2020) among 320 women with chronic pain revealed that psychological inflexibility predicted higher levels of depressive symptoms and sleep disturbance. McCracken et al. (2021) obtained similar findings with 1102 participants in the context of COVID-19. The outcomes established psychological flexibility to be negatively associated with symptoms of depression, anxiety, and insomnia.

Psychological inflexibility is characterized by a rigid pattern of behaviors based on the avoidance of internal experiences (thoughts, feelings, sensations) to the detriment of value-based behaviors (Levin et al., 2014). It occurs through six processes: experiential avoidance, cognitive fusion, lack of awareness of the present moment, self as content, lack of contact with values, and inaction. Psychological inflexibility and experiential avoidance are theorized to contribute to the development, maintenance, and exacerbation of a broad range of psychological problems (Levin et al., 2014), including insomnia. Psychological inflexibility is the focus of acceptance and commitment therapy (ACT) that aims to increase psychological flexibility, that is, the ability to contact the present moment more fully in a way that allows individuals to adjust their behavior more consistently with their goals and values (Hayes et al., 2006).

Sociodemographic, psychiatric, and cognitive factors can influence the severity of insomnia in women, but few studies have been developed to evaluate these characteristics exclusively in this population. Considering the multiplicity of factors involved in the severity of insomnia, knowing the associated factors is essential for the development of targeted intervention strategies.

Given the assumed links among insomnia, sociodemographic, psychiatric, and cognitive factors, this study evaluated the relationship among insomnia severity and sociodemographic variables (age, schooling, and marital status), anxiety complaints, depression, and cognitive variables (beliefs on sleep and psychological inflexibility). Our hypothesis was that the severity of insomnia is associated with low educational level, not being married, increase in age, high anxiety scores, depression, psychological inflexibility, and dysfunctional beliefs about sleep.

## Methods

### Study design and setting

This cross-sectional study incorporated a self-administered survey administered on the Internet. The study was approved by the research ethics committee of the university where it was conducted, and all participants provided informed consent prior to their inclusion. It was managed using REDCap (Research Electronic Data Capture), a secure web platform for building and managing databases and online surveys. This platform allows one to create questionnaire links, consent terms, and store the data collected in a secure web system. The utilization of electronic data capture (EDC) systems is recommended to optimize research data through proper management (Garcia & Abrahão, 2021). REDCap is a web application that can facilitate clinical research development, mainly in the health field, and reduce the costs of conducting research (Garcia & Abrahão, 2021; Walther et al., 2011). Its tools allow researchers to make the best use of EDC components such as data storage.

### Participants and procedure

Our target population for the survey comprised women with insomnia complaints. The participants were recruited from March 2018 to September 2019 through social media and newspaper advertisements. Interested individuals accessed the REDCap database platform and responded to an initial screening, in which the following inclusion criteria were used: (a) being women, b) being 18 to 59 years old, and (c) having insomnia symptoms, as follows: (i) difficulty initiating and/or maintaining sleep, defined as a sleep onset latency and/or wake after sleep onset greater than or equal to 30 min, and waking up earlier than desired with a corresponding sleep time of less than or equal to 6.5 hours per night; (ii) the presence of insomnia for at least 3 nights per week and at least 3 months; (iii) sleep disturbance (or associated daytime fatigue) causing significant distress or impairment in social, occupational, or other areas of functioning. This definition represents a combination of criteria from the American Academy of Sleep Medicine, the *International Classification of Sleep Disorders*, and the *Diagnostic and Statistical Manual of Mental Disorders*, along with quantitative cutoffs typically used in insomnia research (American Academy of Sleep Medicine, 2014; American Psychiatric Association, 2013; Edinger et al., 2004). Those participants who met the inclusion criteria received an email asking them to complete the instruments via the REDCap platform. The data gathered consisted of basic demographic information and standardized questionnaires for insomnia, depression, and anxiety, as well as questionnaires to examine potential cognitive factors such as dysfunctional beliefs about sleep and psychological inflexibility. The participants received no compensation.

### Measures

We used several self-report measures to test the hypotheses of this study. To assess its internal validity—that is, whether we find a good model fit of its original structure with our sample—we ran confirmatory factor analyses (CFAs) for each instrument and calculated reliability coefficients alpha (*α*) and hierarchical omega (*ω*_*h*_). The model’s goodness of fit was assessed using the following fit statistics: chi-squared (*χ*^2^), Tucker-Lewis index (TLI), comparative fit index (CFI), root-mean-square error of approximation (RMSEA), and relative noncentrality Index (RNI). Cutoff values for a good model fit were ⩾ 0.95 for CFI, TLI, and RNI (Hu & Bentler, 1999). For RMSEA, a good fit is indicated by values lower than .06 and no higher than .08, where the upper 90% confidence interval (CI) does not exceed 0.10 (Brown, 2015). We expected values ⩾ 0.70 for adequate internal consistency reliability (Kline, 2015).

### Participants’ data

Sociodemographic information, including age, gender, marital status, and education level, was collected.

### Insomnia Severity Index (ISI)

This instrument was developed by Bastien et al. (2001) and validated in Brazilian Portuguese by Castro (2011). It is a retrospective scale that evaluates the nature, intensity, and impact of insomnia experienced in the last month. Questions are answered using a Likert scale ranging from 0 (no severity) to 4 (high severity) and resulting in a total score from 0 to 28, classified as follows: the absence of insomnia (0–7), mild insomnia (8–14), moderate insomnia (15–21), and severe insomnia (22–28). Originally, Bastien et al. (2001) proposed the ISI as a three-factor model. In the present sample, we achieved a better fit by loading all items in a single factor, compared to the three-factor solution (*Δχ*^2^ = 29.52, *Δ*d.f. = 5, *p* = 0.011), as also reported by previous studies (Ahmed, 2015; Dragioti et al., 2015; Kaufmann et al., 2017). A CFA of the single-factor model produced an acceptable fit [*χ*^2^ (14) = 57.27, *p* < .001; *RMSEA* = 0.086, 95% *CI* [0.066–0.107]; *CFI* = 0.978; *RNI* = 0.978; *TLI* = 0.967]. Internal consistency reliability measures of the observed scale scores were adequate (*α* = 0.698, 95% *CI* [0.656–0.737], ω_*h*_ = 0.706, 95% *CI* [0.666–0.746]).

### Hospital anxiety and depression scale (HADS)

This questionnaire comprises 14 items divided into two subscales, one for anxiety and the other for depression. The global score in each subscale ranges from 0 to 21; the cutoff point most used and recommended by Zigmond and Snaith (1983) ranges from 0 to 8 (anxiety/depression absence) and 9 or greater (anxiety/depression presence). The Brazilian version was initially translated and validated by Botega et al. (1995), with Cronbach’s alpha values of 0.68 and 0.77 for anxiety and depression, respectively. A bifactor model with two group factors (Norton et al., 2013) provided excellent goodness-of-fit indices [*χ*^2^ (60) = 69.2, *p* < .001; *RMSEA* = 0.017, 95% *CI* [0–0.033]; *CFI* = 0.999; *RNI* = 0.999; *TLI* = 0.999]. We observed good reliability indices for the general factor (*α* = 0.87, 95% *CI* [0.851–0.886], ω_*h*_ = 0.87, 95% *CI* [0.851–0.888]).

### Acceptance and action questionnaire-II (AAQ-II)

This retrospective self-report questionnaire was developed by Bond et al. (2011) and evaluates experience avoidance and psychological inflexibility. The items are rated using a scale ranging from 1 (not true) to 7 (always true) such that high scores indicate high experience avoidance. AAQ-II includes items such as “my painful experiences and memories make it difficult for me to live a life that I would value,” “I am afraid of my feelings,” and “I worry about not being able to control my worries and feelings.” The Brazilian version, translated and validated by Barbosa and Murta (2015), demonstrated satisfactory reliability and suitability for study use, both in terms of behavior and therapeutic change. We fitted AAQ-II as a single-factor model with residual correlations between items 1 and 4 as proposed in Bond et al. (2011). We observed an acceptable model fit to the data [*χ*^2^ (13) = 53.15, *p* < .001; *RMSEA* = 0.076, 95% *CI* [0.056–0.098]; *CFI* = 0.998; *RNI* = 0.998; *TLI* = 0.997]. Reliability indices of the general factor indicated high internal consistency (*α* = 0.922, 95% *CI* [0.909–0.932], ω_*h*_ = 0.922, 95% *CI* [0.911–0.934]).

### Dysfunctional beliefs and attitudes about sleep (DBAS-10)

This study used the short version of the DBAS-30 questionnaire, which comprises 10 questions aimed at identifying and evaluating various insomnia-related sleep cognitions (Edinger & Wohlgemuth, 2001; Espie, 2002). We used a translated version produced by the authors of this study since, to date, no Brazilian Portuguese validated version is available. DBAS-10 includes the following items as examples: need 8 h of sleep, need to catch up on sleep loss, consequences of insomnia on health, trying harder will lead to sleep, and insomnia interferes with daytime functioning. The participants were asked to rate each item on a scale ranging from “strongly agree” to “strongly disagree.” The total score is based on the sum of all items and ranges from 0 to 100. A higher score is associated with a higher level of dysfunctional beliefs and attitudes about sleep. For the present sample, we obtained adequate goodness-of-fit indices [*χ*^2^ (32) = 130.29, *p* < .001; *RMSEA* = 0.076, 95% *CI* [0.063–0.09]; *CFI* = 0.973; *RNI* = 0.973; *TLI* = 0.963]. Reliability indices were good for the factor, *immediate negative consequences of insomnia* (*α* = 0.701 95% *CI* [0.646–0.754], ω_*h*_ = 0.69 95% *CI* [0.631–0.748]), reasonable for *long-term negative consequences of insomnia* (*α* = 0.605 95% *CI* [0.532–0.67], ω_*h*_ = 0.626 95% *CI* [0.557–0.694]), and poor for *need for control over insomnia* (*α* = 0.085 95% *CI* [0–0.246], *ω*_*h*_ = 0.365 95% *CI* [0.186–0.544]). For total scores, we found that *α* = 0.74, 95% *CI* [0.67–0.78], and *ω*_*h*_ = 0.74, 95% *CI* [0.70–0.78].

### Data analysis

For each analysis, we included only those participants who provided complete data. Summary statistics, namely frequencies, mean, and standard deviation, were reported for each assessed variable. We described categorical variables as percentages, while continuous variables were expressed in terms of mean and standard deviation. Insomnia severity was classified based on the ISI score, and participants were divided into mild or moderate insomnia (≤ 21 points) and severe insomnia (> 21 points) (Bastien et al., 2001; Castro, 2011). The overall severe insomnia rate was estimated with a 95% CI. Univariate and multivariate analyses were conducted using binary logistic regression models to assess the characteristics associated with severe insomnia. Prior to the analyses, all continuous variables were mean centered to ease interpretation of results. OR and 95% CI were reported for each model. For each independent variable, we estimated an unadjusted OR, resulting from the univariate logistic regression, and an adjusted OR (aOR), resulting from the multivariate logistic regression. The multivariate model included age, marital status (married/not married), educational level (university/no university), anxiety scores (HADS-A cutoff), depression scores (HADS-D cutoff), psychological inflexibility scores (AAQ-II), and dysfunctional beliefs and attitudes about sleep scores (DBAS-10) as predictors of insomnia severity (mild/severe insomnia). To assess model explanatory power, we estimated the coefficient of discrimination (*D*), which is a measure of the difference between the expectations of the distribution of the fitted values for the failures and that of the fitted values for the successes (Tjur, 2009). In short, the *D* measure quantifies the model’s ability to discriminate between successes and failures. The univariate models were fitted with each predictor entered with no covariates. Radar graphs of median values of the AAQ-II and DBAS-10 scales were created to explore their association with the severity of insomnia. All analyses were conducted in the R environment version 4.1.3 (R Core Team, 2022). CFAs were performed using *lavaan* version 0.6.11 (Rosseel, 2012) and the diagonally weighted least squares (DWLS) estimator. Reliability indices were estimated with the *MBESS* package, version 4.9.0 (Kelley, 2022).

## Results

### Sample characteristics

This study included 530 women with an average age of 40.5 years (*SD* = 10.2, range 18–59). Most of the participants had a university degree (65.8%). The overall severe insomnia rate was 182/530 (34.3% [95% *CI* 30.3–38.6]). Table 1 presents the means, standard deviations, and frequencies of the sociodemographic characteristics, scale scores of anxiety, depression, dysfunctional beliefs about sleep, and psychological inflexibility.

**Table 1:** Descriptive analysis of the sample

	*n = 530*
Age (years), mean (SD)	40.5 (10.2)
Marital status	
Married, *n* (%)	252 (47.5)
Not married, *n* (%)	278 (52.5)
Education level	
No university, *n* (%)	117 (25.1)
University, *n* (%)	349 (74.9)
Insomnia Severity Index (ISI) score, mean (SD)	19.6 (4)
≤ 21 points, *n* (%)	348 (65.7)
> 21 points, *n* (%)	182 (34.3)
Anxiety score (HADS-A), mean (SD)	13.0 (4.0)
≤ 8 points, *n* (%)	93 (17.5)
> 8 points, *n* (%)	437 (82.4)
Depression score (HADS-D), mean (SD)	10.8 (4.2)
≤ 8 points, *n* (%)	151 (28.5)
> 8 points, *n* (%)	379 (71.5)
AAQ-II score, mean (SD)	33.2 (9.6)
DBAS-10 score, mean (SD)	79.8 (13.8)

### Factors associated with insomnia severity

As displayed in Table 2, the logistic regression analyses showed that educational level, anxiety scores, depression scores, psychological inflexibility, and dysfunctional beliefs were significantly and positively associated with severe insomnia. The model to predict severe insomnia with age, marital status, educational level, anxiety, depression, psychological inflexibility, and dysfunctional beliefs was statistically significant [*χ*^2^ (8) = 100.43, *p* < .001] and had a moderate explanatory power [*D* = 0.18; Tjur, 2009]. The model’s intercept, corresponding to an observation of being 40.7 years, unmarried, with a university degree, anxiety score ≤ 8, depression score ≤ 8, AAQ-II score of 33.2, and DBAS-10 score of 79.8, was at −1.61 (95% *CI* [−2.46, −0.83], *p* < .001). Within this model, all statistically significant relationships remained except for anxiety scores. Specifically, we found that, holding all other predictor variables constant, the odds for severe insomnia increased by 56% (95% *CI* [1.03, 2.36], *p* = .037) for women without a university degree compared to those with a university degree; 117% (95% *CI* [1.27, 3.81], *p* = 0.006) for women meeting the cutoff for depression compared to those who did not; 5% (95% *CI* [1.02, 1.08], *p* = 0.001) for each additional point on the AAQ-II scale; and 4% (95% *CI* [1.02, 1.06], *p* < 0.001) for each additional point on the DBAS-10 scale.

**Table 2:** Associated sociodemographic factors of severe insomnia and associated cognitive and psychological factors of severe insomnia

	Mild insomnia (*n* = 348)	Severe insomnia (*n* = 182)	Univariate model	Multivariate model
			OR (95% *CI*)	aOR (95% *CI*)
Age (years), mean (SD)	40.9 (10.4)	40.2 (10.0)	0.99 (0.98–1.01)	1.00 (0.98–1.02)
Marital status, *n* (%)				
Married	164 (47.1)	88 (48.4)	-	Reference
Not married	184 (52.9)	94 (51.6)	1.05 (0.73–1.50)	1.03 (0.68–1.54)
Education level, *n* (%)				
University	246 (70.7)	103 (56.6)	-	Reference
No university	102 (29.3)	79 (43.4)	1.85 (1.27–2.69)	1.56 (1.03–2.36)
Anxiety score (HADS-A), *n* (%)				
≤ 8 points	81 (23.3)	12 (6.6)	-	Reference
> 8 points	267 (76.7)	170 (93.4)	4.30 (2.36–8.51)	1.80 (0.98–3.84)
Depression score (HADS-D), *n* (%)				
≤ 8 points	128 (36.8)	23 (12.6)	-	Reference
> 8 points	220 (63.2)	159 (87.4)	4.02 (2.51–6.69)	2.17 (1.27–3.81)
AAQ-II score, mean (SD)	31.0 (9.5)	37.5 (8.3)	1.09 (1.06–1.11)	1.05 (1.02–1.08)
DBAS-10 score, mean (SD)	77.1 (13.8)	85.0 (12.4)	1.05 (1.03–1.07)	1.04 (1.02–1.06)

**Fig. 1:**
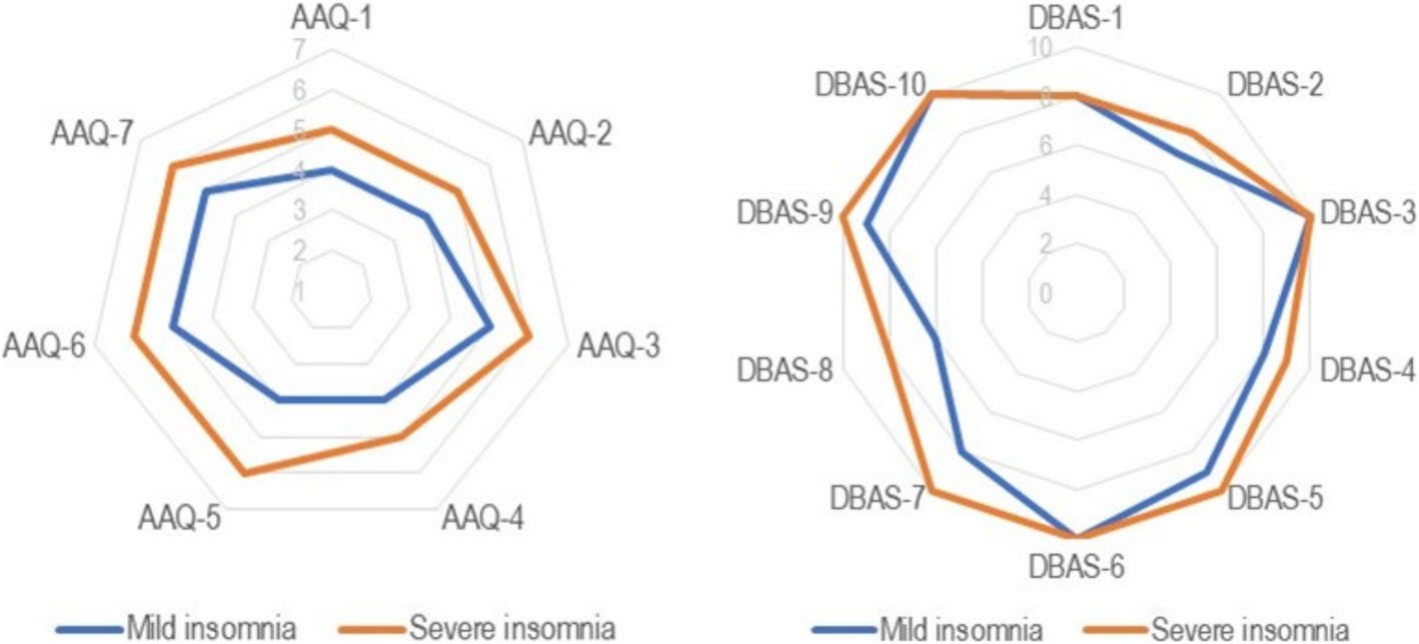
Radar chart of median values of AAQ-II and DBAS-10 scores according to severity of insomnia

Figure 1 illustrates the radar graphs of the AAQ-II and DBAS-10 scales. Women with severe insomnia presented higher scores in all AAQ-II items, especially on “emotions cause problems in my life” (item 5). Although the DBAS-10 total score was higher among women with severe insomnia, we observed equal median values for items 1, 3, 6, and 10 among women with mild/moderate insomnia, which refer to the three factors of beliefs: immediate and long-term negative consequences of insomnia and the need for control over insomnia.

## Discussion

This study evaluated the factors associated with the severity of insomnia in Brazilian women, who reported insomnia complaints. Considering its purpose, additional criteria were employed to quantify the severity of insomnia. For example, difficulty falling asleep is defined as a subjective latency period greater than 20 to 30 min, and difficulty maintaining sleep is defined as a subjective period greater than 20 to 30 min in which the individual remains awake after start sleep. Although there is no standard definition for waking up earlier than usual, this symptom involves waking up at least 30 min earlier than usual and before the total sleep time reaches 6.5 h.

Education level, depression, psychological inflexibility, and dysfunctional beliefs about sleep were associated with an increased rate of severe insomnia complaints, independent of other associated factors. These findings highlight the importance of depression symptoms and cognitive and psychological factors in women’s sleep quality. These results provide support for the notion that insomnia complaints are multifaceted and are associated with both psychiatric and cognitive factors.

Regarding sociodemographic data, our initial hypotheses were partially confirmed. Less schooling (not having a college degree) increased the chance of severe insomnia, and this finding agreed with some of the previously mentioned studies (Hirotsu et al., 2014). Most studies supported this hypothesis under the justification of the association between low education and low income, which would make healthcare difficult (higher prevalence of comorbidities) and result in complex family contexts (double or triple shift, inadequate sleep environment) (Rocha et al., 2002). Our sample comprised 65.8% of women with a university degree, and this was not representative of the Brazilian population, but the size of our sample allowed the results to be reliable.

In contrast with several studies, we found no association between age and insomnia complaints (Torrens et al., 2019). This absence of association may be related to the age of the sample; our study did not include women aged 60 years or older. We believe that over the age of 60, a greater number of comorbidities can worsen sleep and create confounding factors for the individual to differentiate between symptoms. It is important to remember that our sample comprises a particular group of women who already complain of insomnia.

Previous research suggested that not being in a relationship was associated with the presence of insomnia in men and women (Kawata et al., 2020), but our results, exclusively with women, found no association between insomnia severity and being or not being in a relationship. More studies are needed to investigate this finding, considering the different forms of interpersonal and gender relations.

The association between insomnia and depression has been widely reported in literature (Riemann et al., 2019), mainly among women (Morssinkhof et al., 2020), and our findings confirm it. Studies have established that depression or depressive symptoms increase the risk of developing insomnia across multiple age groups (Foley et al., 1999; Morphy et al., 2007), and that insomnia results in nearly two times greater risk for developing depression (Baglioni et al., 2011).

In addition to the characteristics known to be associated with insomnia such as mood, psychological and cognitive factors such as inflexibility and dysfunctional beliefs about sleep, assessed by AAQ-II and DBAS-10 scales, seemed to be related to the severity of insomnia.

Higher AAQ-II scores were associated with higher levels of psychological inflexibility, which, in turn, may serve as a risk factor for psychopathology (Hayes et al., 2012; Kashdan & Rottenberg, 2010). Logistic regression analysis showed that each additional point on the AAQ-II increased the chances of women having severe insomnia. Therefore, our findings are in line with those of previous studies that have reported an association between psychological inflexibility and sleep problems (Daly-Eichenhardt et al., 2016; Kato, 2016, 2020; McCracken et al., 2011).

The differences in the groups were higher in item 5 (emotions cause problems in my life) that addressed emotional avoidance and difficulty in getting in touch with their own emotions. These results are in line with those of previous studies that reported an association between emotional avoidance and the presence and severity of insomnia (Daly-Eichenhardt et al., 2016; McCracken et al., 2011; McCracken et al., 2021; Zakiei et al., 2020).

Authors Lundh (2005) and McCracken et al. (2011) have suggested how the processes of psychological inflexibility/flexibility can affect sleep quality. Emotional avoidance, which refers to the attempt to change the form, frequency, and sensitivity of aversive private events (memories, thoughts, feelings considered negative), can promote increased physiological and cognitive arousal, thus impairing the automatic process of falling asleep (Lundh, 2005). Cognitive fusion, in turn, contributes to an excessive pattern of thoughts and judgments that shape the individual’s relationship with sleep (McCracken et al., 2011). The absence of value-based actions makes daily planning difficult, thereby limiting the individual to a constant pattern of experiential avoidance in relation to sleep difficulties (Lundh, 2005; McCracken et al., 2011). Accordingly, we believe that an experiential avoidance pattern further distances individuals from value-based behaviors, thus contributing to constant dissatisfaction and aggravating insomnia.

To measure psychological inflexibility, we employed the AAQ-II. Walgost (2014) suggested that the AAQ-II measured more psychological distress than psychological inflexibility. As AAQ-II is one-dimensional, it did not allow us to conclude which of the six processes of the psychological inflexibility construct were associated with insomnia complaints (Paulos-Guarnieri et al., 2022).

New instruments have been built to measure the six processes of psychological inflexibility, such as the Multidimensional Psychological Flexibility Inventory (Rolffs et al., 2016) and the comprehensive assessment of acceptance and commitment therapy (Francis et al., 2016). The use of these instruments alongside the AAQ-II can collectively provide a more accurate measure of psychological inflexibility (Ong et al., 2018) and detect which dimensions of psychological inflexibility are most associated with the severity of insomnia. This combination of instruments should be explored in future studies.

DBAS-10 is a widely used questionnaire to detect dysfunctional beliefs about sleep and is also an important follow-up marker for improvement. Our findings demonstrate that each additional point on the DBAS increases the chance of women having severe insomnia. They corroborate previous studies that demonstrated an association between dysfunctional beliefs about sleep and insomnia (Eidelman et al., 2016; Morin et al., 1993), thereby validating the theory of Morin et al. (2007) on the mediating role of cognitions in the exacerbation of insomnia. Dysfunctional beliefs may relate to sleep promotion, the short- and long-term consequences of insomnia, and a lack of control over sleep. Such beliefs can lead to problematic behaviors that can worsen sleep quality, such as staying in bed for extra sleep time, changes in daily routine, cancellation of activities during the day, and feelings of hopelessness (Eidelman et al., 2016; Morin et al., 2002). Interestingly, not all scale items were observed to have higher scores among women with severe insomnia; however, items reporting beliefs about the immediate and long-term negative consequences of insomnia and beliefs about the need for control over insomnia were also reported by women with mild/moderate insomnia.

Our findings that the severity of insomnia is associated with psychological inflexibility and dysfunctional beliefs about sleep provide important contributions to clinical practice; there are therapeutic modalities that focus on reducing dysfunctional beliefs about sleep such as cognitive behavior therapy for insomnia (CBT-I) (Eidelman et al., 2016; Thakral et al., 2020) and on expanding psychological flexibility such as ACT-I (El Rafihi-Ferreira et al., 2020; El Rafihi-Ferreira et al., 2022; Paulos-Guarnieri et al., 2022). Several studies have shown a reduction in dysfunctional beliefs about sleep, with a concomitant reduction in the severity of insomnia (Lance et al., 2019; Thakral et al., 2020). More recently, a reduction in psychological inflexibility and parallel improvement in sleep quality has also been observed in participants with insomnia (Chapoutot et al., 2021; El Rafihi-Ferreira et al., 2020; Paulos-Guarnieri et al., 2022). Interestingly, even though ACT does not have a therapeutic focus on dysfunctional beliefs about sleep, studies have reported reduced DBAS scores after ACT-I-based treatment (El Rafihi-Ferreira et al., 2020; Päivi et al., 2019; Zakiei et al., 2021; Zakiei & Khazaie, 2019). Similarly, El Rafihi-Ferreira et al. (2020) found a reduction in AAQ-II scores after CBT-I treatment, even though CBT did not have a direct focus on psychological flexibility. These outcomes point to the possibility that different therapeutic modalities (contextual or cognitive) lead to effective results in insomnia, including cognitive and psychological variables. Another possibility is that sleep improvement has a positive influence on the psychological aspects; that is, the improvement in psychological flexibility and beliefs is a positive by-product of the sleep improvement observed.

The co-occurrence of depression and insomnia in this sample, especially the association between insomnia severity and higher scores on the depression scale, supports the premise that inflexibility is considered a transdiagnostic process that can occur in different disorders (Levin et al., 2014). Thus, we believe that treatments that focus on psychological flexibility can benefit different disorders that co-occur. Our findings may contribute to the advancement of treatments, including transdiagnostic proposals, by demonstrating that psychological and cognitive factors are involved in the severity of insomnia and co-occur with depression. They also highlight the importance of considering beliefs about sleep and psychological flexibility as therapeutic targets.

A few strengths of our study also deserve mention. We concentrated our analysis only on women, which comprises the majority of insomniac patients. It was important to identify factors associated with insomnia that might be specific for women or manifest differently in both sexes. Moreover, adjusting our analysis for important covariates enabled us to estimate the individual contribution of psychological and cognitive factors.

Nonetheless, our study has its limitations that should warrant care in the interpretation of our findings. First, the use of a Brazilian-Portuguese version of the DBAS-10 has not yet been validated with a representative sample of the target population. Nevertheless, our analysis indicated adequate goodness-of-fit indices for this scale. Second, sleep variables were only assessed with subjective measures. Future studies should also use objective measures of sleep such as actigraphy. This methodology, together with the self-report instruments, would be useful to aid the evaluations, providing greater detail of the sleep characteristics in this population. Third, the absence of a clinical interview does not allow us to discuss the data in terms of the diagnosis of insomnia disorder. Fourth, the associations found in this study may have been confounded by other variables that were not assessed and consequently not adjusted for. The cross-sectional design of the study does not allow us to draw causal conclusions. Longitudinal studies may investigate the direction of the effects found here.

## Conclusion

In summary, our study showed that education level, depression, psychological inflexibility, and dysfunctional beliefs about sleep were associated with severe insomnia. These results are important from a public health perspective because behavioral strategies designed to treat insomnia with a focus on cognitive and psychological factors are low-cost treatments and may help improve sleep quality in women, which also influences mental health. Cohort studies are essential to evaluate the causal effect of education level and cognitive and psychological factors on women’s sleep. Although more studies investigating insomnia severity are needed to better understand their causal relationship with cognitive and psychiatric aspects, these results encourage the development of further behavioral strategies to prevent insomnia severity.

## Data Availability

Data can be requested to the first author by the e-mail address listed in the contact details.

## Abbreviations

AAQ-II: Acceptance and Action Questionnaire-II
ACT: Acceptance and commitment therapy
ACT-I: Acceptance and commitment therapy for insomnia
aOR: Adjusted odds ratio
CBT-I: Cognitive behavioral therapy for insomnia
CFA: Confirmatory factor analysis
CFI: Comparative fit index
CI: Confidence interval
DBAS: Dysfunctional beliefs and attitudes about sleep
DWLS: Diagonally weighted least squares
EDC: Electronic data capture
HADS: Hospital Anxiety and Depression Scale
HADS-A: Hospital Anxiety Scale
HADS-D: Hospital Depression Scale
ISI: Insomnia Severity Index
OR: Odds ratio
REDCap: Research electronic data capture
REM: Rapid eye movement
RMSEA: Root-mean-square error of approximation
RNI: Relative noncentrality index (RNI)
SD: Standard deviation
SWS: Slow wave sleep
TLI: Tucker-Lewis index
WASO: Wake after sleep onset
